# A pilot study of a nurse-led integrated care review (the INCLUDE review) for people with inflammatory rheumatological conditions in primary care: feasibility study findings

**DOI:** 10.1186/s40814-020-00750-7

**Published:** 2021-01-06

**Authors:** Samantha L. Hider, Milica Bucknall, Clare Jinks, Kelly Cooke, Kendra Cooke, Erandie Ediriweera Desilva, Andrew G. Finney, Emma L. Healey, Daniel Herron, Annabelle R. Machin, Christian D. Mallen, Simon Wathall, Carolyn A. Chew-Graham

**Affiliations:** 1 School of Primary, Community and Social Care, Keele University, Keele, Staffordshire ST5 5BG UK; 2 Haywood Academic Rheumatology Centre, Midlands Partnership Foundation Trust, Stoke on Trent, Staffordshire ST6 7AG UK; 3 Keele Clinical Trials Unit, Keele University, Stoke on Trent, UK; 4 National Institute for Health Research (NIHR) Applied Research Collaboration (ARC) West Midlands, Keele, Stoke on Trent, Staffordshire UK; 5 Family Medicine Unit, Faculty of Medicine, University of Colombo, Colombo, Sri Lanka; 6 School of Nursing and Midwifery, Keele University, Clinical Education Centre, University Hospitals of North Midlands NHS Trust, Royal Stoke University Hospital, Stoke-on-Trent, ST4 6QG UK; 7 Midlands Partnership Foundation Trust, Stafford, Staffordshire ST16 3SR UK

**Keywords:** Integrated care, Multimorbidity, Comorbidity, Inflammatory rheumatological conditions, Anxiety, Depression, Cardiovascular disease, Osteoporosis, Long-term conditions

## Abstract

**Background:**

People with inflammatory rheumatological conditions such as rheumatoid arthritis, psoriatic arthritis, ankylosing spondylitis, polymyalgia rheumatica and giant cell arteritis are at an increased risk of common comorbidities including cardiovascular disease, osteoporosis and mood problems, leading to increased morbidity and mortality. Identifying and treating these problems could lead to improved patient quality of life and outcomes. Despite these risks being well-established, patients currently are not systematically targeted for management interventions for these morbidities. This study aimed to assess the feasibility of conducting a randomised controlled trial (RCT) of a nurse-led integrated care review in primary care to identify and manage these morbidities.

**Methods:**

A pilot cluster RCT was delivered across four UK general practices. Patients with a diagnostic Read code for one of the inflammatory rheumatological conditions of interest were recruited by post. In intervention practices (*n* = 2), eligible patients were invited to attend the INCLUDE review. Outcome measures included health-related quality of life (EQ-5D-5L), patient activation, self-efficacy and treatment burden. A sample (*n* = 24) of INCLUDE review consultations were audio-recorded and assessed against a fidelity checklist.

**Results:**

453/789 (57%) patients responded to the invitation, although 114/453 (25%) were excluded as they either did not fulfil eligibility criteria or failed to provide full written consent. In the intervention practices, uptake of the INCLUDE review was high at 72%. Retention at 3 and 6 months both reached pre-specified success criteria. Participants in intervention practices had more primary care contacts than controls (mean 29 vs 22) over the 12 months, with higher prescribing of all relevant medication classes in participants in intervention practices, particularly so for osteoporosis medication (baseline 29% vs 12 month 46%). The intervention was delivered with fidelity, although potential areas for improvement were identified.

**Conclusions:**

The findings of this pilot study suggest it is feasible to deliver an RCT of the nurse-led integrated care (INCLUDE) review in primary care. A significant morbidity burden was identified. Early results suggest the INCLUDE review was associated with changes in practice. Lessons have been learnt around Read codes for patient identification and refining the nurse training.

**Trial registration:**

ISRCTN, ISRCTN12765345

**Supplementary Information:**

The online version contains supplementary material available at 10.1186/s40814-020-00750-7.

## Key messages regarding feasibility

- We were uncertain whether it would be possible to identify patients with inflammatory conditions from primary care records and whether patients with these conditions would attend a nurse-led review.
- More patients than anticipated were identified using primary care records. We found that some patients identified using ankylosing spondylitis Read codes did not report having the condition and a narrower range of codes should be used in any future study.
- It was feasible to recruit and retain patients within the study. Lessons have been learnt around Read codes, refining the nurse training (informed by the fidelity checking), especially around provision of lifestyle advice and the meaning of the case-finding questions for anxiety and depression.

## Background

People with inflammatory rheumatological conditions (IRCs), including rheumatoid arthritis (RA), psoriatic arthritis (PsA), ankylosing spondylitis (AS), polymyalgia rheumatica (PMR) and giant cell arteritis (GCA), are at an increased risk of common comorbidities such as cardiovascular disease (CVD), osteoporosis and depression [1–6], leading to increased morbidity and mortality. This is likely to be due to a combination of lifestyle factors (such as smoking and limited physical activity) plus factors associated with the disease physiology and its treatment. Currently, the identification and management of these comorbidities is fragmented across primary and secondary care, resulting in duplication of some screening or case-finding, such as CVD risk assessment (leading to an inefficient use of resources) [7], whilst other comorbidities (including mood disorders) often remain unrecognised and untreated [8], which has a negative impact on patient outcomes [3, 4]. Data suggests that in patients with RA, these comorbidities are often more severe, but less well managed than in other populations [9]. The National Institute for Health and Care Excellence (NICE) RA guideline advocates an annual review to include assessment of comorbidities [10], although similar guidance does not exist for other inflammatory conditions. For patients with RA, an annual review is incentivised in the Quality and Outcomes Framework [11]; however, the content of this is not specified, meaning important comorbidities could be missed.

Evidence suggests that nurse-delivered reviews are an effective mechanism to identify and manage comorbidity in inflammatory conditions [12] and such an approach reveals a high burden of undiagnosed morbidity [13]. Our hypothesis was that delivering an innovative nurse-led review consultation in primary care could improve patient outcomes and be cost-effective by enabling earlier identification, intervention and management of common comorbidities in people with IRCs. Before testing this new model of care in a definitive trial, we aimed to determine the feasibility and acceptability of this new approach in a two-arm cluster pilot randomised controlled trial (RCT).

## Aims and objectives

The aim of this study was to assess the feasibility of conducting a definitive cluster RCT of a nurse-led holistic integrated care review for people with IRCs in primary care. The primary objectives for this pilot study were as follows:

1. To assess the overall engagement, recruitment and study retention rates of both general practices and participants.
2. To evaluate the intervention uptake and delivery via completion rates of the INCLUDE computer template and the self-reported outcome measures.
3. To assess the fidelity of the intervention delivery by study nurses.
4. To evaluate the acceptability of the integrated care review from the perspectives of practitioners and patients.

The secondary objective was to estimate the variability of key outcomes (as listed in the “Questionnaire outcome measures” section below) to potentially aid in estimation of the sample size and resources required for a future definitive RCT.

## Methods

The study protocol has been published previously [14]. Brief methods for the pilot study are summarised below.

### Recruitment and randomisation

#### Practice recruitment and sample size

As this was a pilot trial, a formal sample size calculation was not required. The initial recruitment target was 100 adults per treatment arm, to ensure that the full range of inflammatory conditions was represented. To achieve this desired final sample size, we estimated that 200 patients would need to be invited per arm, allowing for approximately 50% (*n* = 100) consenting to participate. Previous work using the Consultation in Primary Care Archive (CiPCA) database of primary care medical records suggested that a “typical” general practice with 10,000 patients will have 25, 20, 3 and 4 patients registered with RA, PMR, AS and PsA respectively per year (GCA numbers were not available) [15]. We estimated therefore that between two and six GP practices per arm (depending on practice size and demographics) would be required. Stratified block randomisation was used; stratification was performed by practice size (splitting by order of highest/lowest practice sizes) and block sizes of 2 and/or 4 used within each stratum to ensure balanced cluster and individual patient numbers across treatment arms.

#### Patient recruitment

Practice staff identified an initial list of patients aged 18 years and older who had a diagnostic Read code (a UK coded thesaurus of clinical terms which provide a standardised way of recording diagnoses) for one of the 5 IRCs of interest [rheumatoid arthritis (RA), psoriatic arthritis (PsA), ankylosing spondylitis (AS), polymyalgia rheumatica (PMR) and/or giant cell arteritis (GCA)] in their primary care records. A general practitioner (GP) in each practice reviewed the list for exclusions (e.g. terminal illness, living in nursing home: a full list of inclusions/exclusions is contained within the protocol paper [14]). Patients were mailed a study baseline pack including a Patient Information Sheet and Baseline Questionnaire with consent form. Reminder postcards were sent after 2 weeks and a reminder invitation pack sent after 4 weeks to non-responders. Patients not responding after 4 weeks were considered non-responders. Participants who consented at baseline but did not report having one of the conditions of interest were deemed ineligible for the study and excluded (this appeared to be a particular problem for Read codes for AS; see the “Lessons learnt” section). Patients who did not complete the consent form fully were sent a second letter to confirm missing details—if this was not received after 1 month, the patients were considered ineligible responders and excluded. The study was approved by the Wales REC 5 Research Ethics Committee (REC reference 17/WA/0427). All participants provided written informed consent.

### Study procedures

Responders were sent postal follow-up questionnaires at 3 months and 6 months, with reminder mailings following the same procedure as at baseline. All participants (in both intervention and control arms) received usual GP care for the duration of the study. In consenting participants, medical record review to assess the extent of primary care use was undertaken at 12 months. This was defined as the sum of primary care contacts, which could include face-to-face and telephone consultations, or scanned letters and test results (with several contacts from 1 day counting as a single contact). Furthermore, a rheumatology consultant (lead author SH) reviewed the prescribing records of medications used by participants in this period, a subset of which was subsequently grouped into lipid lowering, antihypertensives, treatments for osteoporosis and antidepressants.

### Questionnaire outcome measures

Full details of the questionnaire measures included are detailed in the protocol paper [14]. Key domains included health-related quality of life (EQ-5D-5L) [16, 17], anxiety (using the Generalised Anxiety Disorder Questionnaire (GAD-7)) [18], depression (using the Patient Health Questionnaire PHQ-8) [19, 20], Patient Activation (PAM) [21], Multimorbidity Treatment Burden (MTBQ) [22] and self-efficacy (Self-Efficacy for Managing Chronic Disease) [23]. Items also measured at follow-up included treatment acceptability and credibility [24], patient satisfaction (using the General Practice Assessment Questionnaire (GPAQ)) [25] and healthcare utilisation. Unfortunately, we were unable to obtain aggregated PAM results from Insignia health and therefore are unable to report these results.

### Control arm

Control participants received usual GP care for the duration of the study.

### Intervention arm

Consenting patients from intervention practices were mailed an INCLUDE intervention appointment letter, giving details of the INCLUDE review appointment at their GP practice (with the option to telephone to rearrange the appointment as required). A reminder telephone call was made approximately 48 h prior to the appointment.

### The INCLUDE review

The content of the INCLUDE review was developed with patients (at two patient advisory groups) and practitioners, and was designed to be a holistic consultation to include case-finding and identification and initial management of CVD (using QRISK2) [26], obesity (assessing body mass index (BMI)), fracture risk (using FRAX) [27], anxiety and depression (using GAD2 and PHQ-2 with full measures (GAD-7 and PHQ-9) used when appropriate) [18–20]. The consultation was recorded by the specialist nurse delivering the INCLUDE review using a study-specific EMIS computer template (the INCLUDE template), which was then saved as part of the patient’s clinical care record within EMIS at the GP study general practice, and then de-identified and securely transferred for analysis. As part of the study template, an individualised management plan was agreed with the patient who was provided with a summary sheet at the end of the review for information. A sample of INCLUDE reviews were digitally recorded, following consent obtained by a study researcher prior to the review consultation.

### Nurse training

The nurses delivering the review (*N* = 2) (who had backgrounds in rheumatology and research) participated in an evidence-based training package delivered over two and a half days, which was designed by clinicians with input from the patient advisory group, to equip them to deliver the review. The training was delivered by members of the research team (CCG and AM). This involved the use of written materials, a slide-set, role play using simulated patients and training to use the GP software system EMIS and the INCLUDE template.

### Process evaluation

A random sample of consultations were digitally recorded (*n* = 24). These were listened to by members of the research team and scored against a pre-specified fidelity checklist, developed by the research team (AM, CCG, EE) (for full checklist, see supplementary table), ensuring that key components of the intervention (around purpose of consultation, physical health measures, cardiovascular risk, fracture risk, mood and signposting for follow-up) had been covered within the consultation. In addition, as part of a mixed methods process evaluation to examine the acceptability of the integrated care review from the perspectives of practitioners and patients participating in the review, a nested qualitative interview study was conducted and is reported separately [28].

### Pilot trial success criteria

We pre-specified a series of criteria to define the success of the pilot trial using criteria adapted from Avery et al. [29], following discussion with the TSC. To fully assess the impact of the INCLUDE intervention, we pre-specified a high retention rate as being key to inform the feasibility and economics of any future large-scale trial. Given that patients were identified in an efficient manner using GP records, it was decided a priori that an uptake rate of 50% would be acceptable. These were based on a Red Amber Green (RAG) rating system and pertained to the following:

1. Response rates: participant response
  Red—Uptake < 25% of eligible patients
  Amber—Uptake 25–50% of eligible patients
  Green—Uptake > 50% of eligible patients
2. Retention rates (at 3 and 6 months)
  Red—Follow-up rate < 50%
  Amber—Follow-up rate 50–70%
  Green—Follow-up rate > 70%
3. Intervention uptake rates (i.e. uptake from invitation to nurse- led review)
  Red—< 30% patients
  Amber—30–50% patients
  Green—> 50% patients

### Statistical analysis

As a pilot study, analysis was exploratory, focused upon process outcomes. Descriptive statistics were used to summarise patient eligibility, recruitment, intervention uptake and retention in the follow-up, overall and stratified by study arm. Self-reported baseline patient data are also summarised by study arm; continuous data were summarised via means and standard deviations or median and interquartile range, depending on data skewness. We estimated means and medians, as appropriate, and associated 95% confidence intervals and interquartile range (IQR) for change between 3 months and 6 months and baseline for each outcome measure, by study arm to determine which outcomes were most sensitive to change over time. Variance component random effects models were used to estimate a range of practice-level intra-cluster correlation coefficients (ICCs), measuring proportion of the individual variance attributable to cluster membership, in order to help inform the sample size calculation for a definitive trial.

Medical records of consenting participants were reviewed for the period between baseline and 12 months follow-up, to assess extent of primary care use. Prescriptions within the first 90 days were considered as baseline (to account for being prescribed medication up to 3 monthly) and compared with those seen at 12 months.

As this study was a feasibility study and not powered to detect differences between arms, *p* values are not presented. All participants were analysed on a randomised complete case basis. Analyses were conducted in Stata 14 (StataCorp LP).

## Results

### Practice recruitment

Twenty-eight general practices across the West Midlands (North) Clinical Research Network region were invited to participate in the INCLUDE study. Of those, 12 practices expressed interest in the study (43%), two practices declined to participate citing involvement in ongoing studies (7%) and 14 practices did not respond to the original invitation to establish interest (50%). Interest was gained from large general practices and there was better than expected recruitment of trial participants to the study, so only practices in randomisation block 1 were included—giving two intervention and two control practices.

### Participant recruitment and retention

**Fig. 1:**
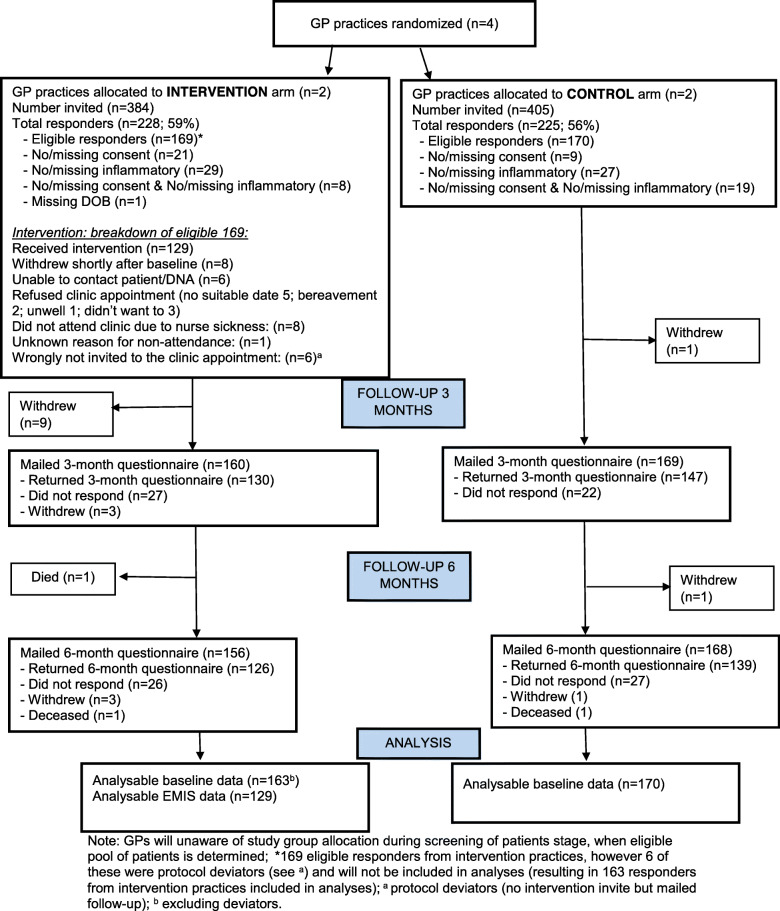
Study participant flowchart

Of the 839 people identified from the practice list screening, 789 (94%) were deemed eligible by their GP and were mailed a baseline study pack. Table 1 details the number of people invited per practice, the response rate and reasons why responders were deemed ineligible. 114/453 (25%) of responders had to be excluded as they either (a) did not provide full written consent, (b) data was missing for IRC or they did not report one of the conditions of interest, or their eligibility based on age could not be determined. The overall response RAG rating was Green at 57%. The overall study flow, consort diagram and reasons for participant exclusion are detailed in Fig. 1.

**Table 1:** Patient recruitment

	Practice population	Identified	Invited after screening	All responders^**1**^	Eligible responders^**2**^	Ineligible responders			Non-responders	Response RAG rating^**3**^
						No/missing consent or DOB	No/missing inflammatory condition	No/missing inflam condition and no/missing consent		
**Intervention**										
Practice A	10,363	208	191	118	91	12	11	4	73	**62% (green)**
Practice C	11,287	195	193	110	78	10	18	4	83	**57% (green)**
**Total**			**384**	**228**	**169**^**4**^	**22**	**29**	**8**	**156**	**59% (green)**
**Control**										
Practice B	9,794	224	212	131	105	6	12	8	81	**62% (green)**
Practice D	10,594	212	193	94	65	3	15	11	99	**49% (amber)**
**Total**			**405**	**225**	**170**	**9**	**27**	**19**	**180**	**56% (green)**
**Overall**		**839**	**789**	**453**	**339**	**31**	**56**	**27**	**336**	**57% (green)**

^1^Sum of eligible responders and ineligible responders

^2^Defined as consent and self-report of one of the inflammatory conditions and age ≥ 18

^3^Calculated as {(baseline eligible responders) + (baseline ineligible responders that consented and did not withdraw)}/invited

^4^Study protocol was deviated for 6 of these participants; hence, they were excluded from the analyses

Table 2 illustrates the participant retention and participation. Questionnaire response rates were Green in both control (56%) and intervention practices (59%). The overall RAG retention rates were over 70% (Green) in all practices at both 3 and 6 months, except one practice had RAG retention rate of Amber (69%) at 6 months. The RAG rating for intervention uptake was Green (> 70%) in both practices. Of the 169 eligible responders in intervention practices, 129 completed the INCLUDE nurse-led intervention (see Fig. 1 for the breakdown). More patients than anticipated were identified with IRCs within each practice, and following discussion to ensure equity and a spread across conditions, we invited all eligible patients within each practice, meaning that we had more participants than anticipated.

**Table 2:** Participant retention

**Intervention**	**Invited**	**Baseline eligible responders**	**Appts completed**	**Uptake RAG rating**^**1**^	**3 months mailed**	**3 months responders**	**3 months retention RAG rating**^**2**^	**6 months mailed**	**6 months responders**	**6 months retention RAG rating**^**3**^
Practice A	191	91	65	71% (green)	85	67	74% (green)	84	63	69% (amber)
Practice C	193	78	64	82% (green)	75	63	81% (green)	72	63	81% (green)
**Total**	**384**	**169**	**129**	**76% (green)**	**160**	**130**	**77% (green)**	**156**	**126**	**75% (green)**
**Control**	**Invited**	**Baseline eligible responders**	**Appts completed**	**Uptake RAG rating**^**2**^	**3 months mailed**	**3 months responders**	**Retention RAG rating**^**2**^	**6 months mailed**	**6 months responders**	**6 months retention RAG rating**^**3**^
Practice B	212	105	N/A	N/A	105	91	87% (green)	104	88	84% (green)
Practice D	193	65	N/A	N/A	64	56	86% (green)	64	51	78% (green)
**Total**	**405**	**170**	N/A	N/A	**169**	**147**	**86% (green)**	**168**	**139**	**82% (green)**
**Overall**	**Invited**	**Baseline eligible responders**	**Appts completed**	**Uptake RAG rating**	**3 months mailed**^**3**^	**3 months responders**	**RAG rating**	**6 months mailed**	**6 months responders**	**6 months retention RAG rating**^**3**^
	**789**	**339**	**129**	**76% (green)**	**329**	**277**	**82% (green)**	**324**	**265**	**78% (green)**

^1^Calculated as appts completed/baseline eligible responders

^2^Calculated as 3 months responders/baseline eligible responders

^3^Calculated as 6 months responders/baseline eligible responders

### Baseline characteristics and follow-up

Table 3 illustrates the baseline characteristics and outcomes of the 333 participants that formed the study sample, overall and stratified by study arm. The mean (SD) age was 68.2 (13.4) years and 200/333 (60%) were female. 319/333 (96%) of patients reported themselves being of white ethnicity. Over half of the participants reported being retired, and 164 (49%) reported ever smoking and a quarter reported consuming alcohol on a daily or almost daily basis. A total of 218 (65%) participants self-reported being overweight (BMI ≥ 25). As was expected, the majority of patients reported either RA (172 (52%)) or PMR (88 (26%)) with 35 (11%) reporting more than one IRC. Nearly half of the patients reported having high blood pressure, and a fifth reported suffering from depression or anxiety.

**Table 3:** Baseline patient characteristics

	All (333)	Control (170)	Intervention (163)
	***N*** (%) *unless otherwise stated*		
**Socio-demographic**			
**Age**, mean (SD)	68.2 (13.4)	67.7 (13.2)	68.6 (13.6)
**Gender**, female	200 (60)	100 (60)	100 (60)
**Ethnicity**, white	319 (96)	165 (97)	154 (94)
**Employment status**			
Employed	68 (20)	31 (18)	37 (23)
Retired	197 (59)	97 (57)	100 (61)
Too ill to work	16 (5)	12 (7)	4 (2)
Unemployed/housewife/househusband	23 (7)	13 (8)	10 (6)
**Lifestyle**			
**Ever smoked**	164 (49)	80 (47)	84 (52)
**Alcohol consumption**			
Daily/3–4 times per week	84 (25)	42 (25)	42 (26)
Once a week/few times per month	116 (35)	60 (35)	56 (34)
Never/special occasions	119 (36)	61 (36)	58 (36)
**BMI**; mean (SD)	26.7 (7.2)	27.3 (7.1)	26.1 (7.3)
**BMI ≥ 25**	218 (65)	111 (65)	107 (66)
**Inflammatory conditions**			
RA	172 (52)	91 (54)	81 (50)
AS	53 (16)	33 (19)	20 (12)
PsA	46 (14)	23 (14)	23 (14)
PMR	88 (26)	44 (26)	44 (27)
GCA	17 (5)	2 (1)	15 (9)
More than one inflammatory condition	35 (11)	20 (12)	15 (9)
**Self-reported health conditions**			
Diabetes	52 (16)	26 (15)	26 (16)
Angina	24 (7)	13 (8)	11 (7)
High blood pressure	154 (46)	80 (47)	74 (45)
Heart attack	25 (8)	13 (8)	12 (7)
Stroke	18 (5)	11 (6)	7 (4)
Depression/anxiety	70 (21)	38 (22)	32 (20)
Osteoporosis	37 (11)	16 (9)	21 (13)
**General health rating**			
Excellent/very good	43 (13)	20 (12)	23 (14)
Good	128 (38)	66 (39)	62 (38)
Fair/poor	154 (46)	78 (46)	76 (47)
**Outcome measures**			
**Current symptoms from inflammatory condition**			
Pain (0–10 NRS), mean (SD) (*n* = 327)	4.9 (2.6)	5.1 (2.7)	4.7 (2.6)
Stiffness (0–10 NRS), mean (SD) (*n* = 326)	5.0 (2.6)	5.4 (2.7)	4.7 (2.5)
Fatigue (0–10 NRS), mean (SD) (*n* = 324)	5.2 (2.7)	5.4 (2.7)	5.0 (2.8)
**Self-Efficacy for Managing Chronic Disease**^**a**^, mean (SD) (*n* = 330)	5.98 (2.52)	5.83 (2.52)	5.98 (2.52)
**General health rating**			
Excellent/very good	43 (13)	20 (12)	23 (14)
Good	128 (38)	66 (39)	62 (38)
Fair/poor	154 (46)	78 (46)	76 (47)
**EQ-5D**^**b**^ **mobility**			
No problems walking	86 (25)	41 (24)	45 (27)
Slight problems walking	99 (30)	49 (29)	50 (31)
Moderate problems walking	106 (32)	57 (33)	49 (30)
Severe problems walking	36 (11)	19 (11)	17 (10)
Unable to walk	2 (1)	1 (1)	1 (1)
Missing	4 (1)	3 (2)	1 (1)
**EQ-5D self-care**			
No problems washing/dressing	196 (59)	98 (58)	98 (60)
Slight problems washing/dressing	68 (20)	31 (18)	37 (23)
Moderate problems washing/dressing	51 (15)	29 (17)	22 (13)
Severe problems washing/dressing	9 (3)	6 (3)	3 (2)
Unable to wash/dress	3 (1)	1 (1)	2 (1)
**EQ-5D usual activities**			
No problems doing usual activities	62 (19)	28 (16)	24 (21)
Slight problems doing usual activities	111 (33)	61 (36)	50 (30)
Moderate problems doing usual activities	108 (33)	51 (30)	57 (35)
Severe problems doing usual activities	37 (11)	22 (13)	15 (9)
Unable to doing usual activities	11 (3)	5 (3)	6 (4)
**EQ-5D pain**			
No pain/discomfort	116 (35)	61 (36)	55 (34)
Slight problems pain/discomfort	134 (40)	65 (39)	69 (42)
Moderate problems pain/discomfort	55 (16)	31 (18)	24 (15)
Severe problems pain/discomfort	4 (1)	2 (1)	2 (1)
Extreme pain/discomfort	5 (2)	4 (2)	1 (1)
**EQ-5D anxiety/depression**			
Not anxious/depressed	150 (45)	79 (47)	71 (43)
Slightly anxious/depressed	111 (33)	55 (32)	56 (34)
Moderately anxious/depressed	57 (17)	28 (17)	29 (18)
Severely anxious/depressed	7 (2)	4 (2)	3 (2)
Extremely anxious/depressed	1 (1)	0 (0)	1 (1)
**FACIT-Fatigue**^**c**^, mean (SD) (*n* = 327)	33.1 (11,6)	33.5 (11.5)	32.7 (11.8)
**Multimorbidity Treatment Burden Questionnaire (MTBQ)**^**d**^			
Median (IQR)	7.5 (2.5, 17.5)	7.5 (2.5, 17.5)	7.5 (2.5, 20)
No burden (score = 0)	72 (22)	37 (22)	35 (21)
Low burden (0 < score < 10)	104 (31)	53 (31)	51 (31)
Medium burden (10 ≥ score < 22)	86 (26)	48 (28)	38 (23)
High burden (22 ≥ score ≤ 100)	67 (20)	29 (17)	38 (23)
**Modified Health Assessment Questionnaire (MHAQ)**^**e**^			
Mild (score ≤ 1.3)	308 (92)	156 (92)	152 (93)
Moderate (1.3 < score ≤ 1.8)	16 (5)	6 (4)	10 (6)
Severe (score > 1.8)	5 (2)	5 (3)	0 (0)
**GAD-7**^**f**^			
Mild anxiety (score 0–5)	208 (62)	110 (65)	98 (60)
Moderate anxiety (6–10)	75 (22)	39 (23)	36 (22)
Moderately severe anxiety (11–15)	36 (11)	16 (9)	20 (12)
Severe anxiety(16–21)	10 (3)	3 (2)	7 (4)
**PHQ-8**^**g**^			
Insignificant depression (score 0–4)	176 (53)	92 (54)	84 (52)
Mild depression (5–9)	86 (26)	44 (26)	42 (26)
Moderate depression (10–14)	39 (12)	17 (10)	22 (13)
Moderately severe depression (15–19)	20 (6)	10 (6)	10 (6)
Severe depression (20–24)	10 (3)	6 (3)	4 (2)

^a^Self-Efficacy for Managing Chronic Disease—composed of 6 items scored 0 (not confident at all) to 10 (totally confident): score calculated as mean of the six items (at least 4 out of 6 items required); lower score indicates worse self-efficacy

^b^EQ-5D: higher score indicates worse health

^c^FACIT-Fatigue—composed of 13 items; scored as 4 = most positive response, 0 = most negative response. Total score calculated as mean of the 13 items (at least 7 out of 13 items required); lower score indicates worse fatigue score

^d^MTBQ—10-item scale: score calculated as mean of the 10 items; higher score indicates worse multimorbidity burden

^e^MHAQ—composed of 8 items scored 0 (without any difficulty) to 3 (unable to do); final score is calculated as the mean of the eight items (at least 6 out of 8 items required); higher score indicates worse function

^f^GAD-7—composed of 7 items scored 0 to 3; score calculated as the total of the six items (score range 0–21); higher score indicates worse anxiety.

^g^PHQ-8—composed of 13 items scored 0–3; score calculated as the total of 13 items (score 0–24); higher score indicates worse depression

Eighty-six (26%) of the 333 participants were lost to either 3-month or 6-month follow-up; there was modest variation between the four practices: 21–34%, with control practices generally having lower loss to follow-up (Table 2). Compared to the 247 participants that responded at both 3- and 6-month follow-up time points, these patients were slightly less likely to be female (58% vs 61%), were on average younger (mean 66 vs 69), but were notably more likely to be employed (28% vs 18%) and less likely to report more than one IRC (7% vs 12%).

### Variability estimates for key outcomes

Table 4 illustrates the variability for the key outcomes over follow-up and between treatment arms. With regard to EQ-5D, MHAQ, GAD and PHQ scores, there were both negligible differences between treatment arms as well as change over time (this is also evident from categorical formation of these measures as presented in Table 3). Control participants reported slightly worse scores of self-efficacy at baseline (mean 5.8 vs 6.0), but by 6-month follow-up, mean score was the same in the two treatment arms. Multimorbidity burden appeared to be approximately the same in the control and intervention arms at baseline (median 7.5 in both); however, whilst the score remained constant over the follow-up among the control participants, it considerably increased among the intervention group (3- and 6-month median = 10). Table 5 details the change in composition of GAD and PHQ categories over time.

**Table 4:** Outcome measures at baseline and follow-up

	Control					Intervention				
	Baseline, mean (SD)	3 months, mean (SD)	6 months, mean (SD)	Mean difference (95% CI): baseline to 3 months	Mean difference (95% CI): baseline to 6 months	Baseline, mean (SD)	3 months, mean (SD)	6 months, mean (SD)	Mean difference (95% CI): baseline to 3 months	Mean difference (95% CI): baseline to 6 months
**Current symptoms from inflammatory condition**										
Pain (0–10 NRS)	5.1 (2.7)	4.8 (2.7)	4.8 (2.6)	− 0.22 (− 0.51, 0.06)	− 0.30 (− 0.62, 0.02)	4.7 (2.6)	4.5 (2.7)	4.6 (2.6)	− 0.13 (− 0.38, 0.12)	− 0.09 (− 0.35, 0.16)
Stiffness (0–10 NRS)	5.4 (2.7)	5.1 (2.6)	5.1 (2.7)	− 0.26 (− 0.53, 0.02)	− 0.25 (− 0.56, 0.05)	4.7 (2.5)	4.7 (2.6)	4.6 (2.6)	0.06 (− 0.18, 0.29)	− 0.13 (− 0.41, 0.16)
Fatigue (0–10 NRS)	5.4 (2.7)	5.1 (2.6)	5.0 (2.6)	− 0.27 (− 0.57, 0.02)	− 0.39 (− 0.67, − 0.10)	5.0 (2.8)	4.9 (2.8)	4.9 (2.7)	− 0.19 (− 0.44, 0.06)	− 0.13 (− 0.42, 0.15)
**Self-Efficacy for Managing Chronic Disease**^**a**^	5.8 (2.5)	NA	6.1 (2.5)	NA	0.25 (− 0.02, 0.51)	6.0 (2.5)	NA	6.1 (2.5)	NA	0.11 (− 0.13, 0.35)
**EQ-5D total**^b^	10.96 (3.69)	10.83 (4.00)	10.60 (3.75)	− 0.17 (− 0.46, 0.12)	− 0.38 (− 0.70, − 0.06)	10.69 (3.62)	10.67 (3.60)	10.72 (3.82)	− 0.01 (− 0.35, 0.32)	0.03 (− 0.29, 0.35)
**FACIT-Fatigue**^**c**^	33.5 (11.5)	NA	27.9 (10.2)	NA	− 5.77 (− 6.75, − 4.79)	32.7 (11.8)	NA	27.6 (10.4)	NA	− 5.01 (− 5.98, − 4.05)
	**Median (IQR)**	**Median (IQR)**	**Median (IQR)**	**Median difference (IQR): baseline to 3 months**	**Median difference (IQR): baseline to 3 months**	**Median (IQR)**	**Median (IQR)**	**Median (IQR)**	**Median difference (IQR): baseline to 3 months**	**Median difference (IQR): baseline to 3 months**
**Multimorbidity Treatment Burden Questionnaire (MTBQ)**^**d**^	7.5 (2.5, 17.5)	7.5 (2.5, 20)	7.5 (2.5, 20)	0 (0, 5)	0 (− 2.5, 5.0)	7.5 (2.5, 20)	10 (2.5, 22.5)	10 (2.5, 20)	0 (− 2.5, 2.5)	0 (− 2.5, 2.5)
**Modified Health Assessment Questionnaire (MHAQ)**^**e**^	0.38 (0, 0.88)	NA	0.38 (0.13, 0.38)	NA	0 (− 0.13, 0.13)	0.38 (0.13, 0.88)	NA	0.38 (0, 0.88)	NA	0 (− 0.13, 0)

^a^Self-Efficacy for Managing Chronic Disease—composed of 6 items scored 0 (not confident at all) to 10 (totally confident): score calculated as mean of the six items (at least 4 out of 6 items required); lower score indicates worse self-efficacy

^b^EQ-5D: higher score indicates worse health

^c^FACIT-Fatigue—composed of 13 items; scored as 4 = most positive response, 0 = most negative response. Total score calculated as mean of the 13 items (at least 7 out of 13 items required); lower score indicates worse fatigue score

^d^MTBQ—10-item scale: score calculated as mean of the 10 items; higher score indicates worse multimorbidity burden

^**e**^MHAQ—composed of 8 items scored 0 (without any difficulty) to 3(unable to do); final score is calculated as the mean of the eight items (at least 6 out of 8 items required); higher score indicates worse function

**Table 5:** GAD and PHQ measures at baseline and follow-up

	All		Control		Intervention	
	Baseline	6 months	Baseline	6 months	Baseline	6 months
**GAD-7**						
Mild anxiety (score 0–5)	208 (62)	173 (52)	110 (65)	93 (55)	98 (60)	80 (49)
Moderate anxiety (6–10)	75 (23)	56 (17)	39 (23)	33 (19)	36 (22)	23 (14)
Moderately severe anxiety (11–15)	36 (11)	21 (6)	16 (9)	10 (6)	20 (12)	11 (7)
Severe anxiety(16–21)	10 (3)	10 (3)	3 (2)	3 (2)	7 (5)	7 (4)
*Missing*	4 (1)	73 (22)	2 (1)	31 (18)	2 (1)	42 (26)
**PHQ-8**						
Insignificant depression (score 0–4)	176 (53)	154 (46)	92 (54)	85 (50)	84 (52)	69 (42)
Mild depression (5–9)	86 (26)	57 (17)	44 (26)	31 (18)	42 (26)	26 (16)
Moderate depression (10–14)	39 (12)	25 (8)	17 (10)	8 (5)	22 (13)	17 (10)
Moderately severe depression (15–19)	20 (6)	15 (5)	10 (6)	10 (6)	10 (6)	5 (3)
Severe depression (20–24)	10 (3)	8 (2)	6 (3)	4 (2)	4 (2)	4 (2)
*Missing*	2 (1)	74 (22)	1 (1)	31 (19)	1 (1)	42 (26)

### Intra-class correlation coefficient

The ICC was calculated using all outcome measures and ranged between 0.003 and 0.04. These ICC estimates however are likely to be imprecise and thus unreliable due to a small number of clusters [30].

### Assessment of the INCLUDE intervention

One hundred twenty-nine out of 169 intervention participants attended the INCLUDE review. The reasons for non-attendance for the remainder of the participants included study withdrawal, inability to contact patient or nurse absence due to sickness, among others (see Fig. 1). The INCLUDE EMIS template was completed for all 129 patients, and the summary information sheet was given to 124 (96%) patients (94% in intervention practice A, 98% in intervention practice C). The recording of individual items, such as pulse, blood pressure, BMI, smoking status and alcohol use, was high (97% overall, 94% in practice A, 100% in practice C).

### Fidelity checking

Whilst most components of the INCLUDE review were delivered as intended in the sample (*n* = 24) of consultations recorded, there were some exceptions (Supplementary Table). Lifestyle advice about weight, diet, activity and alcohol was not, however, always optimally given. In addition, verbal advice was not always backed up with leaflets. There was one example of confusion over FRAX scores, with a calculation being done when a patient was already taking a bisphosphonate, and signposting to the GP not done in one person with a high FRAX score. The meaning of the case-finding questions for anxiety and depression was not always conveyed to the participants fully, but when PHQ-9 and GAD-7 were conducted, the nurses did explain the meaning of the scores. Whilst the written INCLUDE summary sheet was given to patients at the end of each of the recorded reviews, the nurses did not always ask study participants if they had any questions, or respond optimally to queries from patients.

Of the 129 participants who received the intervention, 110 returned the 3-month questionnaire, and therefore, treatment acceptability and credibility, as well as patient satisfaction, at 3 months could be measured for these patients (Table 6). Completion rates for majority of these items were around 75%, which was lower than anticipated. The results identify areas where more attention and emphasis are needed in the training. For example, mean scores were lower for the question that asks “How much improvement in your general health do you think will occur?” This question closely aligns with our hypothesis that patient outcomes can be improved through earlier identification, intervention and management of common comorbidities in people with inflammatory rheumatological conditions (IRCs).

**Table 6:** Treatment acceptability and credibility, and patient satisfaction at 3 months (intervention arm only)

	All participants that received intervention (***n*** = 110^**a**^)	Intervention practice 1	Intervention practice 2
**Treatment acceptability and credibility**^**b**^			
**How logical does the intervention offered to you seem?** Mean (SD); *(% completed item)*	6.3 (3.0); 76%	6.0 (3.0); 76%	6.5 (3.0); 76%
**How successful do you think this intervention will be in improving your health?** Mean (SD); *(% completed item)*	5.3 (2.7); 76%	5.1 (2.8); 78%	5.5 (2.7); 75%
**How confident would you be in recommending this intervention to a friend?** Mean (SD); *(% completed item)*	5 (3.3); 76%	5.2 (3.3); 78%	5.8 (3.4); 75%
**How much improvement in your general health do you think will occur?** Mean (SD); *(% completed item)*	4.2 (3.1); 74%	4.0 (3.4); 76%	4.4 (2.9); 71%
**GPAQ: at your recent INCLUDE review appointment, how good was the nurse at:** ***N*** **(%)**			
**Putting you at ease?**			
Very good	65 (59)	29 (53)	36 (65)
Good	20 (18)	12 (22)	8 (15)
Satisfactory	5 (5)	5 (9)	1 (2)
Poor	0	0	0
Very poor	0	0	0
Missing	19 (17)	9 (16)	10 (18)
**Giving you enough time?**			
Very good	59 (54)	27 (49)	32 (58)
Good	26 (24)	16 (29)	10 (18)
Satisfactory	5 (4)	2 (4)	3 (5)
Poor	1 (1)	1 (2)	0
Very poor	0	0	0
Missing	19 (17)	9 (16)	10 (18)
**Listening to you**			
Very good	58 (53)	26 (47)	32 (58)
Good	24 (22)	14 (25)	10 (18)
Satisfactory	8 (7)	5 (9)	3 (5)
Poor	1 (1)	1 (2)	0 (0)
Very poor	0	0	0 (0)
Missing	19 (17)	9 (16)	10 (18)
**Explaining your condition and treatment?**			
Very good	47 (43)	20 (36)	27 (49)
Good	27 (25)	15 (27)	12 (22)
Satisfactory	7 (6)	4 (7)	3 (5)
Poor	5 (4)	4 (7)	1 (2)
Very poor	0	0	0
Missing	24 (22)	12 (22)	12 (22)
**Involving you in decisions about your care?**			
Very good	40 (36)	15 (27)	25 (45)
Good	30 (27)	17 (31)	13 (24)
Satisfactory	11 (10)	8 (15)	3 (5)
Poor	4 (4)	3 (5)	1 (2)
Very poor	0	0	0 (0)
Missing	25 (23)	12 (22)	13 (24)
**Providing or arranging treatment for you?**			
Very good	39 (35)	14 (25)	25 (45)
Good	26 (24)	14 (25)	12 (22)
Satisfactory	10 (9)	8 (14)	2 (4)
Poor	5 (4)	3 (5)	2 (4)
Very poor	0	0	0
Missing	30 (27)	16 (29)	14 (25)
**Would you be completely happy to see this nurse again?**			
Yes	81 (74)	39 (71)	42 (76
No	6 (5)	4 (7)	2 (4)
Missing	23 (21)	12 (22)	11 (20)

^a^128 participants received intervention, 110 of these completed 3 months questionnaire

^b^All four items are scored as 0 (least) to 10 (most) numerical rating scale

### Medical record review

Two hundred ninety-nine of 333 participants consented to full medical record review (Table 7). The mean number of primary care contacts in the period between baseline and 12-month follow-up was 25 (SD 15). Participants in intervention practices had more primary care contacts (mean 29 vs 22). Over the 12-month follow-up, participants in intervention practices were more likely to be prescribed lipid lowering drugs (51% vs 47%), osteoporosis drugs (46% vs 28%) and antidepressants (25% vs 22%). These observations were less marked in the first 90 days following baseline. A higher proportion of control than intervention participants were prescribed antihypertensive drugs, a finding that was particularly noted in the first 90 days post-baseline (54% vs 46%).

**Table 7:** Medical record review summary

	All (333)	Control (170)	Intervention (163)
Consent to medical record review	299 (90)	157 (92)	142 (87)
Primary care contacts; mean (SD)	25.3 (15.3)	21.9 (13.0)	29.0 (16.8)
**Prescription in 12 months follow-up**			
Cholesterol medication	146 (49)	73 (47)	73 (51)
Antihypertensive medication	171 (57)	92 (59)	79 (56)
Osteoporosis medication	109 (36)	44 (28)	65 (46)
Depression medication	71 (24)	35 (22)	36 (25)
**Prescription in 90 days post-baseline**			
Cholesterol medication	134 (40)	68 (40)	66 (40)
Antihypertensive medication	166 (50)	91 (54)	75 (46)
Osteoporosis medication	82 (25)	35 (21)	47 (29)
Depression medication	59 (18)	31 (18)	28 (17)

### Feasibility criteria

Tables 1 and 2 highlight the results of the pre-specified feasibility criteria. These highlight that the recruitment rates overall reached the Green RAG rating, at 50%. The uptake rates from invitation to nurse-led review in the intervention arm were high at > 70%. The retention rates in the study overall also reached the specified RAG rating of 50%.

## Discussion

The INCLUDE study was designed to assess the feasibility of running a definitive cluster RCT of a nurse-led holistic integrated care review for people with IRCs in primary care. Findings presented here show that the study met its pre-specified success criteria, and hence, it is feasible to deliver a trial of this nature within this population. The recruitment strategy of screening GP records for Read codes of IRCs was successful at identifying a sample of patients who met the eligibility criteria, although the Read codes seemed to over-include patients with AS and lessons have been learnt for future work. The outcome measures were collected successfully at baseline and follow-up, and the INCLUDE intervention and study-specific EMIS template was feasible to use in primary care practice. The intervention was delivered as intended, with most of the components of the intervention delivered with excellent fidelity.

Baseline data showed there was a high burden of self-reported multimorbidity in this population. Whilst there were no major changes between intervention and control practices in the questionnaire outcome measures (although the pilot study was not powered to be able to assess significant differences between groups), there did appear to be differences between the intervention and control patients. Firstly, the self-reported treatment burden was higher in the intervention participants at follow-up, suggesting that additional morbidities were identified and management suggested. Furthermore, practices undertaking these reviews had higher prescribing rates at 12 months (especially of osteoporosis treatments) following treatment of previously un-identified conditions. This suggests that patients with IRCs would benefit from an integrated care review to identify and manage common morbidities.

### Lessons learnt

The results of this pilot study showed that it was feasible to deliver the nurse-led INCLUDE review intervention. There are some limitations with the use of only four GP practices. Whilst initial recruitment rates were excellent, several participants did not report having one of the conditions of interest and it may be that a more stringent/different Read code strategy for inclusion (especially around Read codes for ankylosing spondylitis, AS) would have been of benefit. However, retention rates were excellent at 3 and 6 months.

In terms of intervention delivery and fidelity, analysis of the consultation audio-recordings suggested that consultations were delivered by adhering strictly to the template which sometimes limited explanations of findings, especially around bone health and the use of FRAX. The consultations did not always appear to be patient-centred. These findings have identified where modifications are needed in the training for future interventions.

Acceptability of the INCLUDE review has been investigated in a qualitative interview study (reported elsewhere). We are using the results of the feasibility trial reported here and qualitative study to determine next steps for the INCLUDE review.

## Conclusions

We conclude that it is feasible and practical to deliver a cluster RCT of a nurse-led, holistic, integrated care review for people with IRCs in primary care. Given the impact of managing people with multiple long-term conditions and the comorbidity burden associated with IRCs, such an approach has the potential to improve care and hence morbidity and mortality in this patient group.

## Supplementary Information

**Additional file 1.** INCLUDE fidelity checklist.

## Data Availability

The datasets used and/or analysed during the current study are available from the corresponding author on reasonable request.
