# Supplementary material for: A pilot study of a nurse-led integrated care review (the INCLUDE review) for people with inflammatory rheumatological conditions in primary care: feasibility study findings
Source: Pilot Feasibility Stud. 2021 Jan 6;7:9. doi: 10.1186/s40814-020-00750-7 (PMC7786467; doi:10.1186/s40814-020-00750-7)
Supplement: Supplementary file 1 — Additional file 1. INCLUDE fidelity checklist. [file 40814_2020_750_MOESM1_ESM.docx]

**Checklist for INCLUDE consultation audio recordings**

| **Nurse approach when conducting INCLUDE consultation** | **Demonstration of intervention component** | | | | |
| --- | --- | --- | --- | --- | --- |
|  | **Not applicable** | **Unable to determine from recording** | **No / Not included** | **Somewhat / Partial inclusion** | **Yes / Included as intended** |
| OPENING CONSULTATION | | | | | |
| Explains the review is focussed on health problems linked to their inflammatory rheumatic condition |  |  | 1 | 1 | 22 |
| Explains that people with their condition may have an increased risk of developing cardiovascular disease |  |  | 1 | 1 | 22 |
| Explains people with their condition may have an increased risk of developing osteoporosis |  |  |  | 1 | 23 |
| Explains that many people with their condition may experience low mood/anxiety |  |  |  | 2 | 22 |
| Explains that during the review, they will ask questions to identify whether they could be anxious/ depressed, or if they are at risk of developing osteoporosis/ cardiovascular disease |  |  |  | 2 | 22 |
| Sounds familiar/comfortable with how to access and use the template |  |  |  |  | 24 |
| PHYSICAL HEALTH | | | | | |
| Explains to the patient what their BMI result is and what this means |  |  |  |  | 24 |
| Explores patient’s diet and offers relevant dietary advice |  |  | 4 | 5 | 15 |
| Explores patient’s activity levels and offers relevant exercise advice |  |  | 2 | 2 | 20 |
| Offers weight loss advice, if appropriate | 8 |  | 1 | 5 | 10 |
| Asks the patient about their smoking history |  |  |  |  | 24 |
| Offers smoking cessation advice, if appropriate | 20 |  |  |  | 4 |
| Uses Audit C template to assess alcohol consumption | 2 | 1 |  | 2 | 19 |
| Advises patient about the meaning of their Audit-C score and safe drinking limits | 7 | 1 |  | 4 | 12 |
| CARDIOVASCULAR DISEASE | | | | | |
| Checks patients’ pulse rate and rhythm and offers feedback on these |  |  |  |  | 24 |
| Checks patients’ blood pressure and offers feedback on this |  |  | 1 |  | 23 |
| Reviews cholesterol blood results if available, and advises what they mean | 6 |  |  |  | 18 |
| If patient already on a statin, nurse reviews concordance/ reasons for non-compliance if appropriate | 14 |  |  |  | 10 |
| If calculating QRisk2, checks family history of angina/ heart attack in first degree relative <60yrs first | 13 |  |  |  | 11 |
| Advises what QRisk2 is, and calculates score if appropriate | 13 |  |  | 1 | 10 |
| Advises the patient what QRisk2 score means | 13 |  |  | 1 | 10 |
| OSTEOPOROSIS | | | | | |
| Checks if patient is on treatment for known osteoporosis | 2 |  |  | 1 | 21 |
| If patient has osteoporosis but is not on a bisphosphonate, the nurse determines the reason for this | 18 |  |  |  | 6 |
| If patient has osteoporosis/ GCA or is aged ≥70 yrs with PMR, and not on treatment, nurse advises GP review | 14 | 2 |  |  | 8 |
| If FRAX score indicated, nurse checks if past history of fragility fracture | 10 | 2 |  | 1 | 11 |
| If FRAX score indicated, nurse checks if past history of parental hip fracture | 10 | 2 |  | 2 | 10 |
| If FRAX score indicated, nurse checks if patient currently taking, or previously exposed to >5mg/ day glucocorticoids for >3 months | 10 |  |  | 1 | 13 |
| Advises what FRAX score is | 9 |  | 2 | 2 | 11 |
| Calculates FRAX score and informs patient of score | 9 |  |  | 3 | 12 |
| Offers interpretation of patients’ FRAX score | 9 |  | 2 | 1 | 12 |
| MOOD | | | | | |
| Uses the validated PHQ2 case-finding questions |  |  |  |  | 24 |
| Uses the validated GAD-2 case-finding questions |  |  |  |  | 24 |
| Conveys the meaning of GAD-2 and PHQ2 responses to the patient |  |  | 3 | 13 | 8 |
| Uses validated questions for PHQ-9 | 15 |  |  |  | 9 |
| Uses validated questions for GAD-7 | 14 |  |  |  | 10 |
| Avoids leading prompts that may influence patient response (mood) |  | 1 |  | 1 | 22 |
| Conveys the meaning of GAD-7 and/or PHQ-9 scoring to the patient | 7 |  |  | 1 | 16 |
| MANAGEMENT ADVICE | | | | | |
| Offered EMIS leaflet on healthy lifestyle advice if appropriate | 3 |  | 5 | 9 | 7 |
| Offers resources for management of anxiety/depression (e.g. booklets, phone numbers) | 11 | 1 |  | 2 | 10 |
| Gives self-management advice in relation to mood | 10 |  |  | 2 | 12 |
| FOLLOW-UP | | | | | |
| Suggests GP appointment to review inflammatory rheumatic condition if appropriate | 19 | 1 |  |  | 4 |
| Suggests cholesterol blood test if appropriate | 12 | 3 |  |  | 9 |
| Suggests GP appointment as QRisk >10% and not on a statin | 14 |  |  |  | 10 |
| Suggests practice nurse appointment or home BP monitoring if BP >140/90 and <180/100mmhg | 12 |  | 5 |  | 7 |
| Immediate (duty doc) GP advice sought if BP >180/110mmhg | 21 | 1 |  |  | 2 |
| Suggests ECG and GP follow-up if new irregular pulse detected | 22 |  |  |  | 2 |
| Suggests GP appointment if bisphosphonate indicated and not currently taking | 17 |  |  |  | 6 |
| Suggests GP appointment if on bisphosphonate and no treatment break or DEXA scan over 5 years | 24 |  |  |  |  |
| Advises patient to see their GP to consider requesting a DEXA scan to check for osteoporosis if appropriate | 12 |  |  | 1 | 11 |
| Offered self-referral details for wellbeing/ MIND | 14 |  |  | 2 | 8 |
| Suggests GP appointment if anxious/ depressed and psychological therapies declined, queries about current medication for mood or if patient feels they would benefit from starting a medication for their mood | 17 |  |  |  | 7 |
| Immediate (duty doc) GP advice sought if suicidal risk | 22 |  |  |  | 2 |
| Asks the patient if they have any questions in relation to mood/ CVD or osteoporosis risk |  | 3 | 3 | 12 | 6 |
| If the patient asks questions: Gives suitable response(s) to questions (includes mood, CVD, osteoporosis and research study), consistent with training | 3 |  |  | 6 | 15 |
| Summarises/reaches a shared understanding of the discussion and/or agreed actions |  |  |  | 3 | 21 |
| Uses the summary card to share action plan |  | 1 |  | 1 | 22 |

**Additional Comment:**
